# Effect of *Tripterygium wilfordii* Polycoride on the NOXs-ROS-NLRP3 Inflammasome Signaling Pathway in Mice with Ulcerative Colitis

**DOI:** 10.1155/2019/9306283

**Published:** 2019-08-21

**Authors:** Ma Fangxiao, Ke Yifan, Zhong Jihong, Shen Yan, Liu Yingchao

**Affiliations:** ^1^The Second Clinical Medical College, Zhejiang Chinese Medical University, Hangzhou 310053, China; ^2^Department of Gastroenterology, The Second Affiliated Hospital of Zhejiang Chinese Medical University, Hangzhou 310005, China

## Abstract

**Objective:**

To explore the effect of *Tripterygium wilfordii* polycoride (TWP) on the NADPH oxidases (NOXs)-reactive oxygen species (ROS)-NOD-like receptor protein 3 (NLRP3) inflammasome signaling pathway and the possibility of using TWP to treat ulcerative colitis (UC).

**Methods:**

BALB/c mice were randomly divided into five groups: model control, low TWP, middle TWP, high TWP, and normal control groups. A UC model was established with dextran sulfate sodium. The determination of ROS was carried out by using the fluorescent probe DCFH-DA, and NOXs activity was detected based on the NADPH consumption rate. The mRNA expression levels of NLRP3, ASC, and caspase-1 in the colon tissues and neutrophils were assessed via real-time PCR.

**Results:**

The colon tissues were abnormal with different degrees in TWP groups with disease activity index and histopathological scores lower than those in the model group. In TWP groups, ROS generation, NOXs activity, and the mRNA expression levels of NLRP3, ASC, and caspase-1 in the colon tissues and colon-isolated neutrophils were remarkably lower than those in the model control group (*P* < 0.05) and higher than those in the normal group (*P* < 0.05). The results of pairwise comparison for the efficacy of TWP administration showed that the above indexes were statistically significant with the lowest expression in the high TWP group (*P* < 0.05) and the highest expression in the low TWP group (*P* < 0.05).

**Conclusion:**

TWP demonstrated anti-inflammatory effects on UC by decreasing the expression of proinflammatory factors in the NOXs-ROS-NLRP3 signaling pathway.

## 1. Introduction

Ulcerative colitis (UC) is a type of inflammatory bowel disease (IBD) caused by interactions among multiple factors, including individual susceptibility, environmental causes, food antigens, and intestinal symbiotic bacteria [1, 2]. Currently, UC is considered to be highly associated with an imbalanced immune response. Immune regulation of proinflammatory and anti-inflammatory factors is mediated by intracellular signal mechanisms [3–5], and increases in proinflammatory factors result in a reduction in anti-inflammatory cytokines and an abnormal immune response.

NADPH oxidases (NOXs) are a group of membrane enzymes that generate reactive oxygen species (ROS) in response to infection and pathogens. After stimulation, NOXs activity in immune cells is initiated, which results in ROS production, exhibiting microbicidal activity against infection [6]. NOXs-derived ROS have been shown to mediate the activation of NOD-like receptor protein 3 (NLRP3) inflammasome which is widely distributed in immune cells such as macrophages and T and B lymphocytes [7]. Early studies have reported that NLRP3 inflammasome can induce the production of IL-1*β* and IL-18 and has been associated with the pathogenesis of IBD [8]. Therefore, the NOXs-ROS-NLRP3 inflammasome signaling pathway may have played an important role in the pathogenesis of UC.

*Tripterygium wilfordii* polycoride (TWP) is the main active component of *Tripterygium wilfordii*, which has strong anti-inflammatory and immunomodulatory effects and can inhibit the expression of various cytokines, including IL-1, IL-2, and IL-6 [9]. Previous research has shown that different doses of TWP are effective for treating a UC model established with dextran sulfate sodium (DSS), and the therapy is accompanied by a reduction of NF-*κ*B, IL-1, and TNF-*α* levels [10]. However, its specific mechanism has not yet been fully clarified.

Thus, this study intended to explore the potential mechanisms of UC in relation to NLRP3, and the mechanism by which TWP acts to suppress the inflammatory response was examined.

## 2. Materials and Methods

### 2.1. Animals and Medicine

A total of 50 healthy 8-week-old male mice (SPF) weighing 20 ± 2 g were bought from Zhejiang Chinese Medical University Laboratory Animal Research Center under the animal production license SCXK (Shanghai) 2013-0016. TWP was purchased from Zhejiang DND Pharmaceutical (98% purity, no. Z33020422). The low-, middle-, and high-dose suspensions of TWP were made with distilled water at 9.01, 27.03, and 81.09 mg·kg^−1^, which were converted according to the human-mouse body surface area. DSS was purchased from Sigma-Aldrich (no. 31404, Fluka: 5000 MW). For 5% DSS, 5 mg of DSS was dissolved in 100 mL PBS.

### 2.2. Reagents and Instruments

An animal tissue neutrophil separation kit (Tianjin Haoyang Huake Bioengineering Institute), reactive oxygen species assay kit (Nanjing Jiancheng Bioengineering Institute), NADPH (Beijing Borunlaite Science and Technology Co.), DPI (Sigma-Aldrich), trypsin (Gibco), TRIzol Plus RNA purification kit (Invitrogen), SuperScript III First-Strand Synthesis SuperMix (Invitrogen), Power SYBR Green PCR Master Mix (Applied Biosystems), CFX384 Real-Time PCR System (Bio-Rad), high-speed freezing centrifuge (Sigma-Aldrich), and an ultraviolet spectrophotometer (Beckman) were all purchased as indicated.

### 2.3. Animal Groups and Treatments

As previously described [11], the UC model was established using DSS. We divided the mice into five groups at random: model control, low TWP, middle TWP, high TWP, and normal control groups. In the first week, the mice in the model control, low TWP, middle TWP, and high TWP groups were provided a 5% DSS solution. Meanwhile, the low TWP, middle TWP, and high TWP group mice separately received intragastric administration of 9.01, 27.03, and 81.09 mg/(kg·d) TWP once a day, respectively, with the model control group receiving the same volume of saline. The mice in the normal control group were provided purified water freely, once a day. On day 8, one mouse was randomly sacrificed in each group to observe the general morphology of the colon tissues, and the pathological examination of the colonic tissues (HE staining) was taken to determine whether the modeling was successful. The mice in the five groups drank purified water freely from the beginning of the second week. Additionally, the low TWP, middle TWP, and high TWP group mice were given continual intragastric administration of TWP for 21 days, with the model control group receiving the same volume of saline. Finally, all the mice were sacrificed and the colon tissues with the most significant lesions in all the groups (except the normal control group) were taken for indexes detection and macroscopic and histological examination.

### 2.4. Evaluation of Colitis Severity

We evaluated the disease activity index (DAI) and histology [12]. The characteristics of the stools, hematochezia, and weight of the mice were observed from the beginning of modeling. The success of the modeling was determined by the occurrence of loose stools, hematochezia, weight loss, and morphological changes. The colon tissues were fixed in 10% buffered formalin and 3-4 *μ*m thick sections were prepared and stained with hematoxylin and eosin (H & E). Histological changes were examined with a light microscope. The total DAI score was the sum of the stool, hematochezia, and weight loss scores, and the total histological score was the product of the epithelium and infiltration scores. The scoring criteria are shown in Tables 1 and 2.

### 2.5. Neutrophil Separation from Colon Tissues

Neutrophils from the colon tissues were separated using an animal tissue neutrophil separation kit based on the instructions. The tissue single-cell suspension was prepared by enzymolysis method, and then the neutrophil extract was added and centrifuged. The sedimented neutrophils were removed. The washing solution was added to neutrophils and centrifuged, and then the tissue neutrophils were obtained.

### 2.6. ROS Determination

ROS in the colon tissues and neutrophils was assessed via the reactive oxygen species assay kit based on the instructions. Briefly, the fluorescent probe 2,7-dichlorofluorescein diacetate (DCFH-DA) was added into the cell medium and incubated for 30 min at 37°C in a 5% CO_2_ humidified incubator. And the cells were collected and suspended in PBS after centrifugation. The colon tissues were accurately weighed, and the homogenized medium was added according to the weight (g) : volume (ml) of 1 : 20, which was mechanically homogenized under the condition of ice-water bath. After centrifugation, the supernatant was taken and the DCFH-DA probe was added and incubated for 30 min at 37°C in a 5% CO_2_ humidified incubator. Subsequently, the detection was taken at excitation and emission wavelengths of 500 and 525 nm, respectively, using an ultraviolet spectrophotometer. The results were expressed as fluorescence intensity per mg protein.

### 2.7. NOXs Activity Determination

The colon tissues and neutrophils, digested with trypsin, were suspended in PBS after a 15 min centrifugation (12,000 r/min) at 4°C. A total of 250 *µ*mol/L of NADPH was added to the suspension, which was observed for 5 min at *λ* = 340 nm to determine the consumption of NADPH. NOXs activity was assessed based on the consumption of NADPH after addition of 10 *µ*mol/L DPI (NOXs inhibitor). The absorption extinction coefficient for determining the consumption of NADPH was 6.22 L·mmol^−1^·cm^−1^, and the NOXs activity was expressed in pmol NADPH·min^−1^·mg^−1^.

### 2.8. mRNA Determination for NLRP3, ASC, and Caspase-1

The mRNA expression levels of NLRP3, ASC, and caspase-1 were assessed via real-time PCR. The colon tissues and neutrophils were extracted using TRIzol reagent, and the content and purity of the RNA were determined via ultraviolet spectrophotometry. The solubility curve was analyzed after isolation, followed by qRT-PCR using SuperScript III First-Strand Synthesis SuperMix and RT-PCR Power using SYBR Green PCR Master Mix. The expression levels of mRNA were determined by means of the 2^−ΔΔCt^ method [13]. The sequences of the RT-PCR primers used are shown in Table 3.

### 2.9. Statistical Analysis

Statistical analysis was performed using SPSS 17.0 software, and the results are presented as the mean ± SD. One-way ANOVA and Mann–Whitney *U* tests were used for analysis, and differences were regarded as having statistical significance at *P* < 0.05.

## 3. Results

### 3.1. Effects of TWP on DAI and Histology Scores

Mice in the model control group and the different TWP groups developed symptoms of diarrhea, hematochezia, and weight loss from day 3. However, the above symptoms were significantly alleviated in the different TWP groups compared with the model control group. The normal control group showed normal behavior. Large areas of epithelial crypt loss, prominent neutrophilic infiltration throughout the mucosa, ulceration, and mucosal bleeding were observed in the model control group. In contrast, treatment with TWP resulted in smaller erosions with fewer neutrophils. Erosion, ulceration, and neutrophilic infiltration were not observed in the normal control group. DAI and histological scores of mice in the high TWP group were significantly decreased than those in the model control group (*P* < 0.05). The results are shown in Table 4.

### 3.2. ROS Determination in the Colon Tissues and Neutrophils

**Figure 1:**
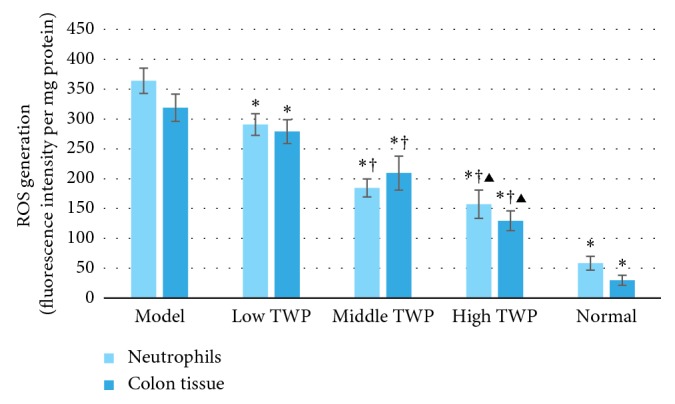
ROS generation in the mouse colon tissues and neutrophils of different groups. Bars of the two colors, respectively, indicate the ROS generation in the mouse colon tissues and neutrophils. Mice in the model control group, low TWP, middle TWP, and high TWP groups were provided a 5% DSS solution for one week. Meanwhile, the low TWP, middle TWP, and high TWP groups received different doses of TWP for 2 weeks. At the end of the experiment, the mouse colon tissues and neutrophils of each group were taken for the ROS content detection via the DCFH-DA (10 mM) fluorescence method. The results showed that the ROS in the colon tissues and neutrophils of the different TWP groups was significantly lower than that of the model control group and higher than that of the normal control group. ROS content decreases with the increased dose of TWP, and the differences among the TWP groups were significant. Each bar represents mean ± SD (*n* = 9); ^*∗*^*P* < 0.05 vs. model; ^†^*P* < 0.05 vs. low TWP; ^▶^*P* < 0.05 vs. middle TWP.

The results of the ROS analysis are shown in Table 5 and Figure 1. The ROS in the colon tissues and neutrophils of the different TWP groups was substantially lower than that of the model control group (*P* < 0.05), whereas it was comparatively higher compared to that of the normal control group (*P* < 0.05). It was also apparent that the ROS content decreased with the increased dose of TWP, and remarkable differences had been observed among the TWP groups (*P* < 0.05).

### 3.3. NOXs Activity in the Colon Tissues and Neutrophils

**Figure 2:**
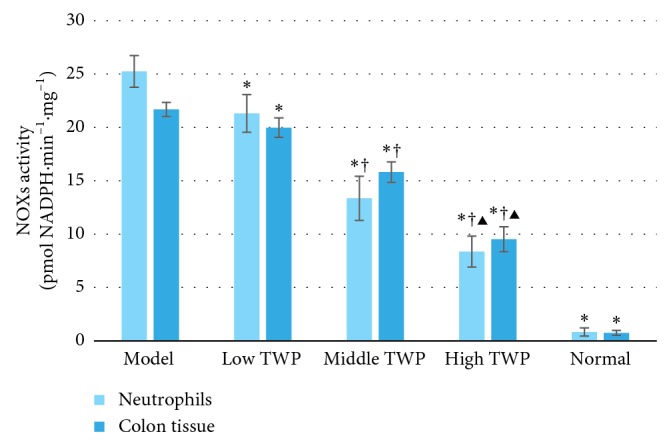
NOXs activity in the colon tissues and neutrophils of different groups. Bars of the two colors, respectively, indicate the NOXs activity in the mouse colon tissues and neutrophils. Mice in the model control, low TWP, middle TWP, and high TWP groups were provided a 5% DSS solution for one week. Meanwhile, the low TWP, middle TWP, and high TWP groups received different doses of TWP for 2 weeks. At the end of the experiment, the mouse colon tissues and neutrophils of each group were taken for the NOXs activity detection based on the NADPH consumption rate. The results showed that NOXs activity in the colon tissues and neutrophils of the different TWP groups was significantly lower than that of the model control group and higher than that of the normal control group. NOXs activity decreases with the increased dose of TWP, and the differences among the TWP groups were significant. Each bar represents mean ± SD (*n* = 9); ^*∗*^*P* < 0.05 vs. model; ^†^*P* < 0.05 vs. low TWP; ^▶^*P* < 0.05 vs. middle TWP.

NOXs activity in the colon tissues and neutrophils of the different TWP groups had a markedly lower expression compared to that of the model control group (*P* < 0.05), whereas it had a significantly higher expression compared to that of the normal control group (*P* < 0.05). In the pairwise comparison of the TWP groups, NOXs activity in the high TWP group was the lowest (*P* < 0.05). In contrast, the NOXs activity in the low TWP group was the highest among the TWP groups (*P* < 0.05). The results are shown in Table 6 and Figure 2.

### 3.4. mRNA Expression of NLRP3, ASC, and Caspase-1

**Figure 3:**
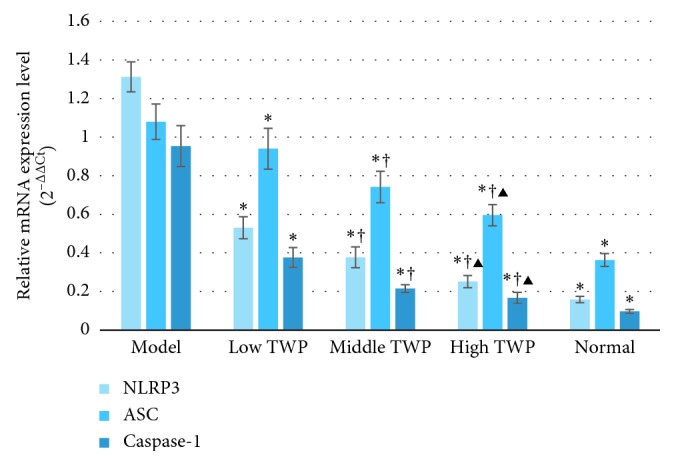
The mRNA expression of NLRP3, ASC, and caspase-1 in neutrophils. Bars of the three colors, respectively, indicate the mRNA expression of NLRP3, ASC, and caspase-1 in neutrophils. Mice in the model control, low TWP, middle TWP, and high TWP groups were provided a 5% DSS solution for one week. Meanwhile, the low TWP, middle TWP, and high TWP groups received different doses of TWP for 2 weeks. At the end of the experiment, the mouse colon neutrophils of each group were taken for the detection of the mRNA expression of NLRP3, ASC, and caspase-1 via real-time PCR. The results showed that the mRNA expression of NLRP3, ASC, and caspase-1 in neutrophils of the different TWP groups was significantly lower than that of the model control group and higher than that of the normal control group. The mRNA expression of NLRP3, ASC, and caspase-1 decreases with the increased dose of TWP, and the differences among the TWP groups were significant. Each bar represents mean ± SD (*n* = 9); ^*∗*^*P* < 0.05 vs. model; ^†^*P* < 0.05 vs. low TWP; ^▶^*P* < 0.05 vs. middle TWP.

**Figure 4:**
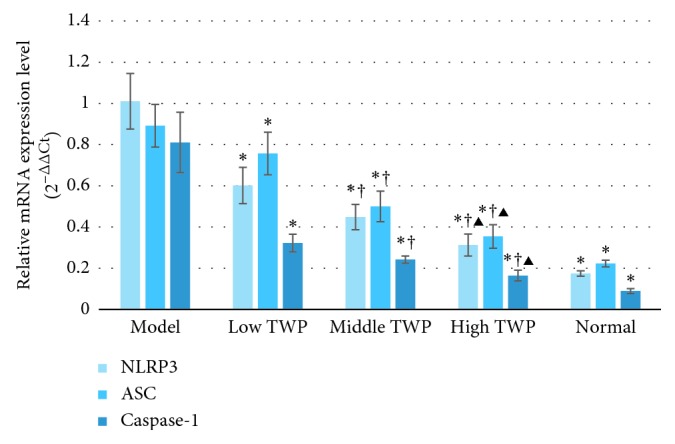
The mRNA expression of NLRP3, ASC, and caspase-1 in the colon tissues. Bars of the three colors, respectively, indicate the mRNA expression of NLRP3, ASC, and caspase-1 in the colon tissues. Mice in the model control, low TWP, middle TWP, and high TWP groups were provided a 5% DSS solution for one week. Meanwhile, the low TWP, middle TWP, and high TWP groups received different doses of TWP for 2 weeks. At the end of the experiment, the mouse colon tissues of each group were taken for the detection of the mRNA expression of NLRP3, ASC, and caspase-1 via real-time PCR. The results showed that the mRNA expression of NLRP3, ASC, and caspase-1 in the colon tissues of the different TWP groups was significantly lower than that of the model control group and higher than that of the normal control group. The mRNA expression of NLRP3, ASC, and caspase-1 decreases with the increased dose of TWP, and the differences among the TWP groups were significant. Each bar represents mean ± SD (*n* = 9); ^*∗*^*P* < 0.05 vs. model; ^†^*P* < 0.05 vs. low TWP; ^▶^*P* < 0.05 vs. middle TWP.

The mRNA expression levels of NLRP3, ASC, and caspase-1 in the colon tissues and neutrophils of the different groups are shown in Figures 3 and 4 . The expression levels of all three mRNAs in the TWP groups were substantially lower compared to that in the model control group (*P* < 0.05) and higher than that in the normal control group (*P* < 0.05). The levels of NLRP3, ASC, and caspase-1 in the colon tissues and neutrophils decreased with the increased dose of TWP among the TWP groups (*P* < 0.05) (Tables 7 and 8).

## 4. Discussion

UC is a chronic nonspecific colitis that is characterized by its protracted course of disease. Moreover, the incidence of carcinogenesis in patients with UC for 10–20 years is as high as 28% [14]. In recent years, the incidence of UC has shown an increasing trend with a tendency to occur in younger patients than previously documented. It is commonly accepted that UC is a result of genetic predisposition, infectivity, immune disorders, psychosomatic disease, and autoimmune disease, but the pathogenesis of UC is not entirely clear [15, 16]. To date, western medical treatments include 5-aminosalicylic acid, glucocorticoid, immunosuppressants, and newer biological preparations, all of which have side effects and poor efficacies when used over extended periods. Thus, it is imperative to identify new treatments for UC with fewer side effects and that are suitable for long-term use.

TWP, an aqueous-chloroform extract from *Tripterygium wilfordii*, has strong anti-inflammatory and immunomodulatory effects. TWP has demonstrated a good therapeutic effect on UC by inhibiting various cytokines, such as IL-1, IL-2, IL-4, IL-5, IL-6, IL-8, IL-12, and TNF-*α* [17–19].

The NLRP3 inflammasome, a multiprotein complex, consists of NLRP3, ASC, and caspase-1 [20]. The connection between the NLRP3 PYD domain and the ASC PYD domain is accomplished by the activation of NLRP3. The CARD domain of ASC then combines with caspase-1, which mediates the activation of caspase-1. As it has been reported, the NLRP3 inflammasome has an extremely essential role in the innate immune system, which can be activated by the pathogen-associated molecular patterns and damage-associated molecular patterns [21, 22]. Recently, massive levels of researchers have studied the effects of the NLRP3 inflammasome on the immune pathogenesis of UC. In 2010, the study by Zaki et al. [23] showed that the susceptibility to colitis induced by oral 3% DSS and TNBS enema was clearly increased in NLRP3-, ASC-, and caspase-1-deficient mice, which suggested that the NLRP3 inflammasome was extremely critical to the maintenance of the intestinal homeostasis. Bauer et al. [24] also found that the tolerance of colitis induced by DSS in NLRP3-deficient mice was likely associated with the reduction of the colonic proinflammatory cytokines IL-l*β*, IL-18, and TNF-*α*.

As is known, the NLRP3 inflammasome can be activated via the following three mechanisms: (1) potassium efflux, (2) lysosome damage, and (3) generation of ROS. The generation of ROS is the main activation mechanism for the NLRP3 inflammasome, a majority of which comes from the action of NOXs in the intestinal mucosa [25]. Under physiological conditions, ROS contributes to the protection of the intestine from invading microbes. However, overexpression of NOXs can be caused by various factors, including pathogenic microorganisms, and this can result in an increase in ROS production. Excessive ROS helps to activate nuclear factors involved in producing proinflammatory cytokines, thus promoting the inflammatory response [26, 27]. NOXs are expressed in the gastrointestinal epithelium mucosa, macrophages, and neutrophils, and neutrophils directly participate in the inflammatory reaction via their ability to migrate to and aggregate at specific sites [28, 29]. As such, the pathogenesis of UC is possibly linked to the NLRP3 inflammasome being activated by ROS and the subsequent induction of inflammatory cytokine release. Subsequently, the NF-*κ*B pathway is activated, and the inflammatory response is boosted further.

The results of this study indicated that the activity of NOXs in the colon tissues and neutrophils demonstrated a significant rise in the UC groups, which suggested that NOXs not only help maintain physiological functions but that they also have a clear relationship with the increase in inflammatory cells that results in UC. NOXs are located within the cell at a resting state in the absence of ROS. However, NOXs can be translocated to the surface of the cell and quickly activated by ROS in response to external stimuli, including pathogenic microorganisms and cytokines. Massive levels of ROS can contribute to the activation of multiple transcription factors, which could induce gene transcription and aggravate the inflammatory response [30, 31]. In addition, the obviously increased mRNA expression of NLRP3, ASC, and caspase-1 in the model groups illustrated that the signaling pathway associated with the NLRP3 inflammasome played a vital role in UC. And the increase of NLRP3, ASC, and caspase-1 that are included in this signaling pathway bears a close relation to the development of UC. An activated NLRP3 inflammasome can recruit enough procaspase-1 through ASC and result in the hydrolysis of two adjacent procaspase-1 to produce caspase-1 with enzyme activity, which promotes the activation of IL-1*β*, thereby enhancing the inflammatory reaction of the colonic epithelial cells and resulting in UC [32].

## 5. Conclusions

The ROS levels, NOXs activity, and the expression levels of NLRP3, ASC, and caspase-1 in the colon tissues and neutrophils of the TWP groups were lower compared to those of the model control group, suggesting that TWP could inhibit NOXs activity and ROS production. Subsequently, the activation of the NLRP3 inflammasome, ASC, and caspase-1 would be restrained, followed by a reduction of proinflammatory factors. Together, these observations indicated that TWP had anti-inflammatory effects on UC by way of inhibiting the NOXs-ROS-NLRP3 signaling pathway. In conclusion, TWP has considerable prospects for application in the treatment of UC. Additionally, components of the NLRP3 inflammasome have the potential to serve as novel targets for the treatment of UC.

## Figures and Tables

**Table 1:** The criteria of DAI scores.

Score	Stool	Hematochezia	Weight loss (%)
0	Normal form	Occult blood negative	0
1			1–5
2	Loose and not forming	Occult blood positive	5–10
3			10–15
4	Watery stool	Eye blood	>15

**Table 2:** The criteria of histological scores.

Features	Scores	Description
Epithelium	0	Normal morphology
	1	Loss of goblet cells
	2	Loss of goblet cells in large areas
	3	Loss of crypts
	4	Loss of crypts in large areas

Infiltration	0	No infiltration
	1	Infiltration around crypt bases
	2	Infiltration reaching the muscularis mucosa
	3	Extensive infiltration reaching the muscularis mucosa and thickening of the mucosa with abundant edema
	4	Infiltration of the submucosa

**Table 3:** The sequences of the RT-PCR primers.

Name	GenBank accession	Primer sequences (5′-3′)	Size (bp)	Annealing (°C)
Rat NLRP3	NM_001191642.1	AAGCAGCAGATGGAGACTGGAAA TGAACAGAGCCCTGGCAGGTAG	149	63
Rat caspase-1	NM_012762.2	TGAACAAAGAAGGTGGCGCATT GGCAAGACGTGTACGAGTGGGT	140	63
Mouse ASC	NM_023258.4	GGTCACAGAAGTGGACGGAGTG CATCTTGTCTTGGCTGGTGGTCT	103	63
Mouse 18s	NR_003278	CGGACACGGACAGGATTGACA CCAGACAAATCGCTCCACCAACTA	94	63

**Table 4:** The scores of DAI and histology (mean ± SD, *n* = 9).

Group	DAI scores	Histological scores
Model control group	1.51 ± 0.88	5.18 ± 2.27
Low TWP group	0.80 ± 0.77	3.33 ± 1.29
Middle TWP group	0.61 ± 0.58	2.85 ± 1.32
High TWP group	0.47 ± 0.46^*∗*^	2.36 ± 1.54^*∗*^
Normal control group	0.06 ± 0.05	0.92 ± 0.90

Note: compared with the model control group, ^*∗*^*P* < 0.05.

**Table 5:** ROS determination in the colon tissues and neutrophils (fluorescence intensity per mg protein) (mean ± SD, *n* = 9).

Group	Neutrophils	Colon tissues
Model control group	364.09 ± 21.25	319.04 ± 22.84
Low TWP group	290.79 ± 18.20^*∗*^	279.01 ± 19.92^*∗*^
Middle TWP group	184.66 ± 15.01^*∗*^^†^	209.52 ± 28.43^*∗*^^†^
High TWP group	157.27 ± 23.65^*∗*^^†^^▶^	129.52 ± 16.59^*∗*^^†^^▶^
Normal control group	58.49 ± 11.70^*∗*^	29.95 ± 8.41^*∗*^

Note: ^*∗*^*P* < 0.05 vs. model; ^†^*P* < 0.05 vs. low TWP; ^▶^*P* < 0.05 vs. middle TWP.

**Table 6:** NOXs activity in the colon tissues and neutrophils (pmol·NADPH·min^−1^·mg^−1^) (mean ± SD, *n* = 9).

Group	Neutrophils	Colon tissues
Model control group	25.25 ± 1.48	21.69 ± 0.66
Low TWP group	21.32 ± 1.76^*∗*^	19.98 ± 0.91^*∗*^
Middle TWP group	13.36 ± 2.07^*∗*^^†^	15.82 ± 0.96^*∗*^^†^
High TWP group	8.37 ± 1.45^*∗*^^†^^▶^	9.53 ± 1.17^*∗*^^†^^▶^
Normal control group	0.84 ± 0.38^*∗*^	0.75 ± 0.23^*∗*^

Note: ^*∗*^*P* < 0.05 vs. model; ^†^*P* < 0.05 vs. low TWP; ^▶^*P* < 0.05 vs. middle TWP.

**Table 7:** The mRNA expression of NLRP3, ASC, and caspase-1 in neutrophils (2^−ΔΔCt^) (mean ± SD, *n* = 9).

Group	NLRP3	ASC	Caspase-1
Model control group	1.31 ± 0.08	1.08 ± 0.09	1.00 ± 0.10
Low TWP group	0.53 ± 0.06^*∗*^	0.94 ± 0.11^*∗*^	0.38 ± 0.05^*∗*^
Middle TWP group	0.38 ± 0.05^*∗*^^†^	0.74 ± 0.08^*∗*^^†^	0.22 ± 0.02^*∗*^^†^
High TWP group	0.25 ± 0.03^*∗*^^†^^▶^	0.60 ± 0.05^*∗*^^†^^▶^	0.17 ± 0.03^*∗*^^†^^▶^
Normal control group	0.16 ± 0.02^*∗*^	0.36 ± 0.03^*∗*^	0.10 ± 0.01^*∗*^

Note: ^*∗*^*P* < 0.05 vs. model; ^†^*P* < 0.05 vs. low TWP; ^▶^*P* < 0.05 vs. middle TWP.

**Table 8:** The mRNA expression of NLRP3, ASC, and caspase-1 in the colon tissues (2^−ΔΔCt^) (mean ± SD, *n* = 9).

Group	NLRP3	ASC	Caspase-1
Model control group	1.01 ± 0.13	0.89 ± 0.10	0.81 ± 0.15
Low TWP group	0.60 ± 0.09^*∗*^	0.76 ± 0.10^*∗*^	0.32 ± 0.04^*∗*^
Middle TWP group	0.45 ± 0.06^*∗*^^†^	0.50 ± 0.07^*∗*^^†^	0.24 ± 0.02^*∗*^^†^
High TWP group	0.31 ± 0.05^*∗*^^†^^▶^	0.35 ± 0.06^*∗*^^†^^▶^	0.16 ± 0.03^*∗*^^†^^▶^
Normal control group	0.17 ± 0.01^*∗*^	0.22 ± 0.02^*∗*^	0.09 ± 0.01^*∗*^

Note: ^*∗*^*P* < 0.05 vs. model; ^†^*P* < 0.05 vs. low TWP; ^▶^*P* < 0.05 vs. middle TWP.

## Data Availability

The data used to support the findings of this study are available from the corresponding author upon request.
